# Social and environmental stressors of cardiometabolic health

**DOI:** 10.1038/s41598-024-64847-2

**Published:** 2024-06-19

**Authors:** Anna Bartoskova Polcrova, Andrea Dalecka, Daniel Szabo, Juan Pablo Gonzalez Rivas, Martin Bobak, Hynek Pikhart

**Affiliations:** 1 RECETOX, Faculty of Science, Masaryk University, Kotlarska 2, Brno, Czech Republic; 2 International Clinical Research Centre (ICRC), St Anne’s University Hospital Brno (FNUSA), Brno, Czech Republic; 3 Department of Global Health and Population, Harvard TH Chan School of Public Health, Harvard University, Boston, MA USA; 4 Department of Epidemiology and Public Health, University College London, London, UK

**Keywords:** Environmental social sciences, Risk factors

## Abstract

Exposures to social and environmental stressors arise individual behavioural response and thus indirectly affect cardiometabolic health. The aim of this study was to investigate several social and environmental stressors and the paths of their influence on cardiometabolic health. The data of 2154 participants (aged 25–64 years) from the cross-sectional population-based study were analysed. The composite score of metabolic disorders (MS score) was calculated based on 5 biomarkers: waist circumference, blood pressure, fasting blood glucose, HDL-cholesterol, triglycerides. The effects of social stressors (education level, income), environmental stressors (NO_2_, noise) and behavioural factors (unhealthy diet, smoking, alcohol consumption, sedentary behaviours) on MS score were assessed using a structural model. We observed a direct effect of education on MS score, as well as an indirect effect mediated via an unhealthy diet, smoking, and sedentary behaviours. We also observed a significant indirect effect of income via sedentary behaviours. The only environmental stressor predicting MS was noise, which also mediated the effect of education. In summary, the effect of social stressors on the development of cardiometabolic risk had a higher magnitude than the effect of the assessed environmental factors. Social stressors lead to an individual’s unhealthy behaviour and might predispose individuals to higher levels of environmental stressors exposures.

## Introduction

Cardiovascular diseases (CVD) are globally responsible for the biggest proportion of deaths. In 2019^1^, there were approximately 523 million cases of CVD and 19 million CVD deaths worldwide. In Europe, cardiovascular mortality in the last three decades slightly decreased, from 48% in 1990 to 44% in 2019. The drop was even more noticeable in Czechia, where the proportion of deaths caused by CVD decreased from 55% in 1990 to 45% in 2019^2^. The reduction of cardiovascular mortality can be explained by the improvements in healthcare after the socio-political transformation in the early 90s^3^ as well as positive changes in the individual lifestyle^4,5^. Although the CVD mortality rate declined in recent years, the burden of cardiometabolic drivers such as abnormal adiposity and dysglycaemia in the population increased^2^. Cardiometabolic health is influenced by a complex network of social and environmental stressors^6^, as demonstrated by the ubiquitous health inequalities in human populations. This complex system of exposures, acting on the background of the human genome, influences morbidity and mortality risk, and it has been recently included in so-called exposome approach to disease^7^. The exposome concept represents the overall impact of diverse factors on human health and consists of both, external and internal factors^8^.

The social environment is formed by a complex network of social and economic conditions such as level of education, income, financial deprivation, occupation, social status as well as neighbourhood or cultural characteristics^9^. People from disadvantaged environments experience higher rates of poor health and disabilities and, therefore, are at a higher risk of premature death^9–11^. Previous studies also reported significant associations between lower socioeconomic position and increased CVD incidence and mortality^12,13^. Similarly, in a previous study including 8449 subjects from Czechia^10^, the level of education was identified as the strongest determinant of cardiovascular mortality, followed by hypertension and smoking^10^. Socioeconomic disadvantages also predispose individuals to increased external stressors exposure including negative life events, lack of resources, life insecurity, limited access to health care, or environmental stressors exposure^14,15^.

The environmental stressors represent characteristics of the built environment together with the natural condition of the living areas as well as physical and chemical pollution. The built environment consists of aspects built by humans such as urban spaces, access to greenspace, transportation, walkways, etc. Previous studies suggested that living in more walkable, less sprawled areas^16^ and having good access to greenspace^17^, are associated with a lower risk of obesity, type 2 diabetes, and hypertension. Physical and chemical pollution refers to short-term and long-term exposures to environmental factors that mostly result from various human activities such as industry, power plants, transportation, or household activities. Air pollution, noise, and heavy metal emissions belong among the most important environmental hazards that affect public health^18^.

Air pollution is characterized by exposure to PM_2.5_, PM_10,_ and NO_2,_ which has been previously associated with adverse cardiometabolic health including dysglycaemia^19^ and hypertension^20^. The mixed evidence about the relationship between air pollution exposure and adiposity was observed. In a systematic review from 2018, An et al. described that only 56% of assessed studies found a significant relationship between adiposity and air pollution, with the association being positive in 44% of studies and negative in 12%^21^. The main sources of PM_2.5_, PM_10,_ and NO_2_ in urban environment is traffic, which is also a major source of another environmental stressor—noise.

The effect of long-term noise exposure on diverse cardiometabolic health biomarkers has been reported. There is considerable evidence about the association between excessive noise exposure and hypertension^22,23^ and type 2 diabetes^24^. Traffic noise has been positively associated also with higher BMI and waist circumference, although the effects were generally small and less consistent^25–29^.

It is important to consider, that all factors including social and environmental stressors are interconnected. In particular, socioeconomic disadvantage may trigger exposure to other external risk factors^9^. Similarly, the exposure to social and environmental stressors arises behavioural response and thus indirectly affects health^30^.

The impact of stressors is even enhanced by their cumulation^31^ and a wide range of their interactions and pathways. The exposome approach thus offers the concept of complex exposure assessment which can help to identify the pathways by which stressors affect human health and allow us to better understand the aetiology of chronic diseases^7,8,32^. Despite the large amount of previous literature focused of social and environmental stressors, there is still need for extension of the evidence focused on exposome concept in the exposure assessment. Most of the reported literature focus on the assessment of the individual effect of risk factors, but evidence including a comprehensive view of the entire exposome is limited. For a successful strategy of declining inequalities, a deep understanding of social and environmental stressors and their influence is needed. This study aims to model structural relations between social and environmental stressors and cardiometabolic health.

## Methods

### Design and population

Data from the Kardiovize study^33^ were used. The Kardiovize study is an epidemiological study including a random sample of adult residents (aged 25 to 65 years) of the city of Brno, the second-largest city in Czechia, with 373,327 residents. Survey sampling was done in January 2013 with technical assistance from the health insurance companies. A random age and sex-stratified sample of 2154 men and women has been enrolled in the study. No information on non-respondents was available due to confidentiality restrictions.

### Data collection

In-person health interviews were performed by trained nurses and physicians at the International Clinical Research Center of the St Anne’s University Hospital in Brno. The questionnaire included demographics, socioeconomic characteristics, cardiovascular risk behaviours, smoking status, medical history, and mental health. The geocode for the living location has been obtained at the street level for 2157 participants and at the district level for 71 participants.

### Measures

#### Cardiometabolic risk

Five cardiometabolic biomarkers were assessed. Waist circumference was measured using manual tape. Blood pressure was measured with the participant alone using an automated office measurement device (BpTRU, model BPM 200; Bp TRU Medical Devices Ltd., Canada). Three measurements were performed and averaged. Laboratory analyses were performed with12-hour fasting full blood samples. The composite score of cardiometabolic risk was calculated based on the presence of metabolic syndrome components. The components of metabolic syndrome were assessed based on the previous definitions^34^: (1) waist circumference > 94 cm in men or > 80 cm in women; (2) systolic blood pressure > 130 mmHg or diastolic blood pressure > 85 mmHg or the reported use of antihypertensive medication; (3) fasting blood glucose > 5.6 mmol/l or the reported use of antidiabetic medication; (4) HDL-cholesterol < 1.0 mmol/l in men or < 1.3 mmol/l in women or reported use of hypolipidemic medication; (5) triglycerides > 1.7 mmol/l or reported use of hypolipidemic medication. The five components were summed up, and the MS score was created, ranging from 0 to 5 points, with higher scores representing higher cardiometabolic risk.

#### Social stressors

##### Education

Educational attainment was classified into three groups: “high”, including subjects with higher professional or university education, where higher professional qualification refers to specialized training beyond secondary education, leading to recognized certification or licensure for specific occupations; “middle”, defined as high school education with a final graduation exam; and “low”, defined as elementary or vocational education without a final graduation exam.

##### Income

Self-reported household income was assessed in the equalized form to consider the differences in a household’s size and composition. Data about total household income were collected using categories defined by income ranges. The mid value of each range was then used. The equivalized household income was calculated as a ratio of total household income and equivalent size. The equivalent size is calculated by attributing a weight to all members of household in following way: 1.0 for the first person and 0.5 for each subsequent person in the household. The equivalent size is the sum of the weights of all the members of a given household^35^.

#### Environmental stressors

##### Air pollution

To assess the effect of air pollution on cardiometabolic risk, nitrogen dioxide (NO_2_) exposure was included in the model. For the complete assessment of the effect of air pollution on cardiometabolic health, it would be desirable to also consider the effect of PM_10_ and PM_2.5_, however, the variance in their exposure was insufficient, with an interquartile range of 2.90 and 3.75 μg/m^3^, respectively. Similar variance has been observed in previous study from Brno^36^.

5 year mean NO_2_ concentrations for the years 2008–2012 were obtained from air pollution level maps of Czech Hydrometeorological Institute at a spatial resolution of 1 × 1 km^37^. The pollution maps are interpolated on annual basis from a combination of measured air pollution data, several models of dispersion (primarily CAMx, SYMOS and EMEP), traffic emissions, elevation, and population density (see Škáchová and Vlasáková^38^ for more details). Ground-level NO_2_ concentrations were obtained for each residential building at its centroid, and mean, median and standard deviation values of residential buildings’ concentrations were obtained for each street. For the addresses geocoded on the street level, the mean values of residential buildings’ concentrations were used. For the addresses geocoded at the district level, air pollution levels were imputed from 50 buildings nearest to the district centroid.

##### Noise

The environmental noise exposures were obtained from the results of the prediction model of the 2nd report on Strategic noise mapping in the Czech Republic (2012), conducted in accordance with the environmental noise directive (END) requirements and methods^39^. Global combined (road, railway, and airport) day-evening-night noise levels (L_den)_ were calculated for each residential building at its centroid, and mean, median and standard deviation values were obtained for each street. Missing data in the noise prediction model within the borders of the modeled territory were imputed with the lowest category of the noise level. For the addresses geocoded at the district level, noise levels were imputed from 50 buildings nearest to the district centroid.

#### Behavioural factors

##### Dietary risk

Dietary risky patterns were assessed using a dietary risk score derived from the 43-item food frequency questionnaire (FFQ). Participants were asked to indicate the frequency of consumption of specific food groups in the past week on a scale including 10 options from “almost never” to “six or more times a day”. In total, six specific risky dietary patterns were identified based on the global burden of disease (GBD)^40^ methodology (Table 1). The occurrences of each risky dietary patterns were summed, so the total dietary risk score ranged from 0 to 6 points.

**Table 1:** Definition of dietary risky score items.

Diet low in fruit	Mean daily consumption of fruits (fresh, frozen, cooked, canned, or dried fruits, excluding fruit juices and salted or pickled fruits)	Less than 250 g per day
Diet low in vegetables	Mean daily consumption of vegetables (fresh, frozen, cooked, canned, or dried vegetables, excluding legumes and salted or pickled vegetables, juices, nuts, seeds, and starchy vegetables such as potatoes or corn)	Less than 360 g per day
Diet high in red meat	Mean daily consumption of red meat (beef, pork, lamb, and goat, but excluding poultry, fish, eggs, and all processed meats)	More than 23 g per day
Diet high in processed meat	Mean daily consumption of meat preserved by smoking, curing, salting, or addition of chemical preservatives	More than 2 g per day
Diet low in nuts and seeds	Mean daily consumption of nut and seed foods	Less than 21 g per day
Diet low in legumes	Mean daily consumption of legumes (fresh, frozen, cooked, canned, or dried legumes)	Less than 60 g per day

##### Smoking

Smoking status was assessed using the self-report method and categorized as current smokers, ex-smokers, and non-smokers.

##### Alcohol intake

Alcohol intake was evaluated as the self-reported total amount of ethanol (derived from reported amount of beer, wine and spirits) in grams consumed during the week before data collection.

##### Sedentary behaviours

Sedentary behaviours were based on total sitting time in minutes per week, obtained from the long version of the international questionnaire of physical activity^41^ (IPAQ).

### Data analysis

Data analyses were performed using STATA^42^ software (version 16.0, StataCorp, College Station, TX, USA) and MPlus 8.6^43^.Continuous variables were described using means, and categorical variables using frequencies. The Ordinal regression was performed to assess the association between social or environmental factors and cardiometabolic risk score. General structural equation modeling was implemented to describe the pathways and structural relationships between the stressors, and between the stressors and outcome. We constructed structural model with social factors as independent variables determining behavioural factors as well as environmental exposures, and cardiometabolic risk as the main assessed outcome, predicted by social factors directly but also indirectly. Thus, we tested the direct effects of social and environmental stressors on cardiometabolic risk as well as the indirect effects of social stressors through behavioural and environmental mediators. All variables were ordered from the lowest value (the lowest category for ordinal variables) to the highest. All tested associations were further adjusted for sex and age. P values less than 0.05 were considered statistically significant. A complete case analysis method was used for handling missing data.

### Ethical statements

The study protocol complied with the Helsinki declaration and all participants signed the informed consent. The study was approved by the ethical committee of St Anne’s University Hospital, Brno, Czech Republic on 13 June 2012 (reference number 2 G/2012).

## Results

### Subjects’ characteristics

In total, 2154 (54.7% women) subjects were included in the analysis with a mean age of 47.29 years. The descriptive characteristics are shown in Table 2. The most prevalent level of education was high (41.60%). The exposure concentrations to NO_2_ ranged from 7.80 to 42.30 μg/m^3^. The noise exposure ranged from 42.50 to 66.97 dB.

**Table 2:** Descriptive characteristics of the analytical sample.

n	2154 (54.64% women)
Age (years) (mean ± SD)	47.29 ± 11.29
Prevalence of metabolic syndrome components (%)	
High waist circumference	54.22
High blood pressure	43.27
High blood glucose	14.21
Low HDL	19.59
High triglycerides	27.44
Presence of MS components (%)	
0	29.57
1	24.05
2	20.66
3	13.09
4	9.05
5	3.57
Average metabolic syndrome score (mean ± SD)	1.59 ± 1.44
Social stressors	
Income (CZK)	20,802.54 ± 11,720.26
Education (%)	
Low	19.82
Middle	38.58
High	41.60
Environmental stressors	
NO_2_ (μg/m^3^) (mean ± SD)	24.89 ± 6.31
Noise (dB) (mean ± SD)	53.79 ± 4.06
Behavioral factors	
Dietary risk score (0 (healthy)—6 (risky)) (mean ± SD)	4.69 ± 1.07
Sedentary behavior (min)^a^ (mean ± SD)	2894.04 ± 1210.24
Alcohol consumption (g)^b^ (mean ± SD)	78.11 ± 99.12
Smoking (%)	
Smokers	23.46
Ex-smokers	25.45
Non-smokers	51.09

*MS* metabolic syndrome.

^a^Reported in total sitting time in minutes per week. ^b^Grams of ethanol consumed in the last 7 days.

### The association between stressors and cardiometabolic risk

We tested the total effect of each social, environmental, and behavioural factor on MS score in a separate model using ordinal regression analysis. The results showed that better socioeconomic condition was associated with lower risk of increased MS score. Those with high education level showed 52% lower odds of having higher MS score compared to those with low education level and by every 10,000CZK increase in household income, the odds of higher MS score decreased by 11% (Table 3).

**Table 3:** The associations between social, environmental, and behavioural factors and MS in ordinal regression analysis.

		OR	p	95% CI
Education	Low	1 (ref)		
	Middle	0.81	0.053	0.65 to 1.00
	High	0.48	< 0.001	0.39 to 0.60
Income	Per 10,000CZK	0.89	0.001	0.83 to 0.95
NO_2_	Per 10 μg/m^3^	1.11	0.085	0.99 to 1.26
Noise	Per 10 dB	1.34	0.004	1.10 to 1.63
Dietary risk	Per 1 unit of score	1.24	< 0.001	1.15 to 1.34
Sedentary	Per 100 min	1.01	< 0.001	1.01 to 1.02
Alcohol	Per 10 g	1.00	0.343	0.99 to 1.01
Smoking	Non-smokers	1 (ref)		
	Ex-smokers	1.31	0.006	1.08 to 1.58
	Smokers	1.46	< 0.001	1.21 to 1.77

The OR indicate the odds of one level higher MS.

*MS* metabolic syndrome score.

Our results also indicated 34% increase odds of higher MS score with every 10 dB increase in environmental noise exposure. Among behavioural factors, we identified significantly increased odds of higher MS score with increased dietary risk, higher sedentary time and in smokers or ex-smokers compared to non-smokers (Table 3).

### The structural model of stressors, behavioural factors, and cardiometabolic risk

**Figure 1:**
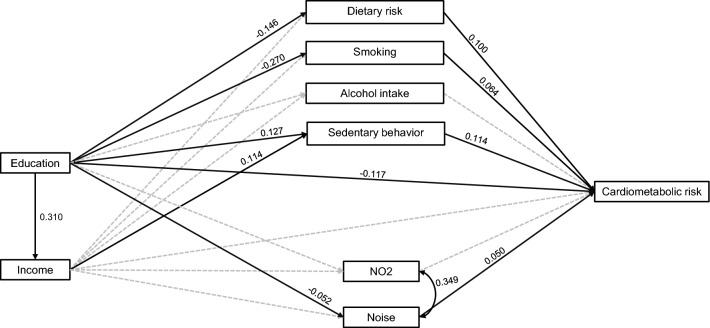
Observed statistically significant relationships in complex structural model. Bold values represent direct and the indirect effect through specific mediator. Reported results significant at p < 0.05. Dotted lines represent tested but statistically non-significant relationships (at 5% level of significance).

The results of structural model showed that higher level of education significantly predicted increased income and decreased dietary risk and decreased smoking. However, at the same time, higher education was associated with increased sedentary behaviours (Table 4). The only environmental stressor significantly predicting higher MS score was noise (β = 0.050; 95% CI [0.004, 0.092]) (Fig. 1, Table 4).

**Table 4:** Estimated relationships between all variables in the structural model.

Predictor		Outcome	β	p	95% CI
Education	→	MS	** − 0.117**	** < 0.001**	** − 0.161 to − 0.073**
	→	Income	**0.310**	** < 0.001**	**0.272 to 0.344**
	→	Dietary risk	** − 0.146**	** < 0.001**	** − 0.192 to − 0.100**
	→	Sedentary	**0.127**	** < 0.001**	**0.083 to 0.172**
	→	Alcohol	0.014	0.533	** − **0.028 to 0.060
	→	Smoking	** − 0.270**	** < 0.001**	** − 0.318 to − 0.223**
	→	NO_2_	** − **0.010	0.654	** − **0.054 to 0.035
	→	Noise	** − **0.052	0.027	** − **0.100 to 0.023
Income	→	MS	** − **0.044	0.066	** − **0.090 to 0.005
	→	Dietary risk	** − **0.035	0.144	** − **0.080 to 0.013
	→	Sedentary	**0.114**	** < 0.001**	**0.069 to 0.161**
	→	Alcohol	0.045	0.081	** − **0.004 to 0.099
	→	Smoking	0.019	0.509	** − **0.041 to 0.075
	→	NO_2_	− 0.022	0.337	** − **0.067 to 0.023
	→	Noise	− 0.043	0.106	** − **0.092 to 0.012
NO_2_	→	MS	0.015	0.503	** − **0.029 to 0.057
Noise	→	MS	**0.050**	**0.027**	**0.004 to 0.092**
Dietary risk	→	MS	**0.100**	** < 0.001**	**0.059 to 0.147**
Sedentary	→	MS	**0.114**	** < 0.001**	**0.077 to 0.153**
Alcohol	→	MS	0.024	0.263	** − **0.018 to 0.067
Smoking	→	MS	**0.064**	**0.007**	**0.019 to 0.114**

The reference categories are low education and low income. The β indicates standardized regression coefficient. Model fit: χ^2^(14) = 94.79, p < 0.001, CFI = 0.954, RMSEA = 0.052, 90% CI RMSEA [0.042, 0.062].

*MS* metabolic syndrome score. *CFI* comparative fit index, *RMSEA* root mean square error of approximation.

Significant values are in bold.

We observed a significant direct effect of higher education on MS score (β =  − 0.117; 95% CI [− 0.161, − 0.073], as well as an indirect effect mediated via dietary risk (β =  − 0.0153; 95% CI [− 0.024, − 0.008]), smoking (β =  − 0.017; 95% CI [− 0.032, − 0.005]), sedentary behaviours (β = 0.014; 95% CI [0.008, 0.024]) and noise exposure (β =  − 0.003; 95% CI [− 0.007, − 0.001]). We also observed significant indirect effect via the path including income and sedentary behaviours as successive mediators. The total mediation ratio was 21.0% (Table 5). Although we did not observe a statistically significant direct effect of income on MS score, the results showed a significant indirect effect of higher income via sedentary behaviours (β = 0.013; 95% CI [0.007, 0.021]) (Table 5).

**Table 5:** Standardized total, direct, and indirect effects of mediators in the association between education or income and cardiometabolic risk observed in structural model*.

	Total effect		Direct effect		Indirect effect		
	β	95% CI	β	95% CI		β	95% CI
Education	− **0.147**	− **0.185 to** − **0.108**	− **0.117**	− **0.161 to** − **0.073**	Total	− **0.031**	− **0.053 to** − **0.008**
Partial through listed mediators					Income	− 0.014	− 0.028 to 0.001
					Dietary risk	− **0.015**	− **0.024 to -0.008**
					Smoking	− **0.017**	− **0.032 to** − **0.005**
					Alcohol consumption	0.000	**0.000 to 0.003**
					Sedentary behaviour	**0.014**	**0.008 to 0.024**
					NO_2_	0.000	− 0.003 to 0.001
					Noise	− **0.003**	− **0.007 to** − **0.001**
					Income—Dietary risk	− 0.001	− 0.003 to 0.000
					Income—Smoking	0.000	− 0.001 to 0.002
					Income—Alcohol	**0.000**	**0.000 to 0.002**
					Income—Sedentary	**0.004**	**0.002 to 0.007**
					Income—NO_2_	0.000	− 0.001 to 0.000
					Income—Noise	− 0.001	− 0.002 to 0.000
Income	− 0.034	− 0.079 to 0.014	− 0.044	− 0.090 to 0.005	Total	0.009	− 0.001 to 0.021
Partial through listed mediators					Dietary risk	− 0.004	− 0.010 to 0.001
					Smoking	0.001	− 0.002 to 0.006
					Alcohol consumption	**0.001**	**0.000 to 0.005**
					Sedentary behaviour	**0.013**	**0.007 to 0.021**
					NO_2_	0.000	− 0.003 to 0.001
					Noise	− 0.002	− 0.007 to 0.000

*Results adjusted for sex and age.

Significant values are in bold.

As a sensitivity analysis, we run the sex-stratified analysis (Supplementary Table 1). In men, we observed slightly higher effect of smoking and lower effect of dietary risk on MS score, compared to women. Similarly, in women, we observed higher effect of income and lower effect of education on sedentary, compared to men. In men and women, the overall indirect effects were − 0.040 (p = 0.032) and − 0.037 (p = 0.017) with a mediation ratio of 25.3% and 26.8%, respectively. In general, the overall differences between men and women are small, therefore we included the full results in supplementary materials.

## Discussion

The purpose of this study was to investigate the network of social and environmental stressors and the paths of their effect on cardiometabolic risk. Lower level of education was associated with increased cardiometabolic risk but also with smoking and unhealthy dietary patterns as well as increased exposure to environmental noise, which all together contribute to cardiometabolic risk. On the contrary, higher levels of education was associated with increased sedentary behaviours, also associated with increased cardiometabolic risk. Sedentary behaviours, therefore, potentially decreased the protective effect of higher education on cardiometabolic risk. Additionally, sedentary behaviour was identified as a significant mediator of increased cardiometabolic risk in individuals with higher income though income itself was not directly associated with cardiometabolic risk.

The direct effect of social determinants on health is driven by physiological responses to stress, arising from disadvantageous life environment. People with disadvantaged socioeconomic position exhibit more physiological stress^44^ which therefore lead to the internal dysregulation and increased cardiometabolic risk.

The indirect effect of social determinants may be mediated through several pathways. In our study, we investigated the mediating role of behavioural factors and environmental exposures. The increased prevalence of inappropriate lifestyle in socio-economically disadvantaged group has been previously described. According to previous literature, socio-economically disadvantaged groups develop and exhibit more unhealthy behaviours, such as tobacco use, excessive alcohol use, physical inactivity, and poor nutrition^45^. At the same time, lower education may be reflected in reduced knowledge-related skills and limited health literacy^46^, which all together again trigger unhealthy behaviours. Additionally, according to the previous studies, disadvantaged populations live in less prestigious neighbourhoods with limited resources that may be reflected for instance in lower availability of sport facilities and greenspaces^47–49^, increased exposure to unhealthy diet options^47,50–53^ and higher environmental pollution^15^. Therefore, we can assume that unhealthy behaviour as cardiometabolic risk factors arise from socio-economic disadvantage and at the same time, behavioural response may partially explain social inequalities in cardiometabolic health. However, we cannot neglect the role of sedentary behaviour, which is, on the contrary, a cardiometabolic risk factor linked to socioeconomic advantage. The increasing prevalence of sedentary behaviours in recent years^54^ could in the future lead to increase of burden of cardiometabolic risk in higher socioeconomic groups.

The second investigated path included environmental exposure as mediators of the effect of social determinants on cardiometabolic risk. Previous studies reported that socioeconomic disadvantage may predispose individuals to increased environmental exposures^9^. The United States Environmental Protection Agency (US EPA) acknowledged that that environmental exposures are considered as an additional health burden to these disadvantaged groups. Several societies have addressed this issue by adopting specific plans to strengthen environmental justice^55^. We investigated the role of long-term air pollution exposure (NO_2_) and environmental noise exposure. Our results showed no significant association between long-term NO2 exposure on cardiometabolic risk. Even though the exposures have been historically relatively low in city of Brno (IQR = 20.40–29.95) compared to capitols and big cities in Europe, the concentrations still exceeded the Air Quality Guideline (annual NO_2_ = 10 μg/m^3^) recommended by WHO in 2021. On the other hand, the effects of low-level exposures on cardiometabolic outcomes have been generally very weak in previous studies. For instance, a study of the Dutch national health survey represented by more than 380,000 adults showed that NO_2_ exposure predicted only 6% odds (OR = 1.06; 95% CI 1.04–1.09) of diabetes and 2% odds (OR = 1.06; 95% CI 1.04–1.09) for hypertension^56^. Therefore, we assume that our inconclusive results might be caused by relatively low study power as well as low exposure variance in the area.

The environmental noise was the only environmental stressor identified as mediator of the association between social determinants and cardiometabolic risk. The effect of long-term noise exposure on cardiometabolic health has been previously investigated. There is evidence about the association between excessive noise exposure and hypertension^22,23^, as well as type 2 diabetes^24^ and waist circumference^25^. Previous studies also suggested several underlying mechanisms of reported associations. Environmental noise exposure influence haemostasis and vascular function and incites oxidative stress as well as systematic inflammation^57^. Long-term environmental noise exposure also causes sleep deprivation which may lead to other physiological or psychological consequences^57^. Based on our results, we may assume that noise exposure associated with urban life environment is another explanation of social inequalities in cardiometabolic health.

The major strength of the present study is the complex approach of cardiometabolic risk assessment, including multiple measures of cardiometabolic health. Also, we examined a wide spectrum of healthy behaviour risk factors as well as two important environmental exposures. Furthermore, we included confounding and mediation analyses that contributed to reveal important interplay mechanisms between socioeconomic, behavioural and environmental stressors of cardiometabolic health. However, there are some limitations of this study that deserve to be mentioned. First, the cross-sectional design of the study does not allow for evaluating causality, thus the direction of the associations set in the structural model was constructed based on previous evidence, and reverse causation bias might occur. Second, we had no information about the year of onset of risky levels of cardiometabolic biomarkers, therefore, we may not be sure whether exposures precede the heath outcome. Third, the residential mobility of the participants may lead to under- or over-estimation of the exposure levels. Forth, study sample probably did not provide enough study power to reveal a significant association between air pollution exposure and cardiometabolic risk. Moreover, occupational exposures have not been considered due to data unavailability. Fifth, the study sample only included a city-based population; thus, the study findings should not be generalized beyond the urban population. Additionally, the study population included only White Europeans, thereby limiting the generalizability of the findings for other ethnicities.

## Conclusion

This study highlights the intricate network of social and environmental stressors and their impact on cardiometabolic risk. Lower levels of education were found to directly increase cardiometabolic risk while also predisposing individuals to unhealthy behaviours such as smoking and poor dietary patterns. Additionally, lower education levels were associated with increased exposure to environmental noise, further contributing to cardiometabolic risk. On the other hand, higher levels of education and income were linked to increased sedentary behaviours, which diminished the protective potential against cardiometabolic risk. The findings emphasize the role of both physiological responses to stress and behavioural factors in the direct and indirect effects of social determinants on health. Moreover, environmental exposures, particularly long-term noise exposure, were identified as mediators of the association between social determinants and cardiometabolic risk. Nevertheless, this study underscores the importance of addressing social inequalities and environmental factors to improve public health outcomes related to cardiometabolic risk.

### Supplementary Information

Supplementary Table 1.

## Data Availability

The data that support the findings of this study are available from ICRC—FNUSA but restrictions apply to the availability of these data, which were used under license for the current study, and so are not publicly available. Data are available from Juan Pablo Gonzalez Rivas upon reasonable request and with permission of ICRC-FNUSA.
