# Supplementary material for: Social and environmental stressors of cardiometabolic health
Source: Sci Rep. 2024 Jun 19;14:14179. doi: 10.1038/s41598-024-64847-2 (PMC11187061; doi:10.1038/s41598-024-64847-2)
Supplement: Supplementary file 1 — Supplementary Table 1. [file 41598_2024_64847_MOESM1_ESM.docx]

| **Supplementary table 1: Estimated relationships between all variables in the structural model stratified by sex.** | | | | | | |
| --- | --- | --- | --- | --- | --- | --- |
|  |  |  | **Men** | | **Women** | |
| **Predictor** |  | **Outcome** | **β** | **95% CI** | **β** | **95% CI** |
| Education | → | MS | **-0.118** | **-0.181 to -0.053** | **-0.101** | **-0.162 to -0.043** |
|  | → | Income | **0.299** | **0.246 to 0.348** | **0.335** | **0.284 to 0.383** |
|  | → | Dietary risk | **-0.141** | **-0.203 to -0.064** | **-0.159** | **-0.221 to -0.092** |
|  | → | Sedentary | **0.224** | **0.158 to 0.290** | 0.033 | -0.031 to 0.099 |
|  | → | Alcohol | -0.018 | -0.086 to 0.050 | **0.071** | **0.008 to 0.131** |
|  | → | Smoking | **-0.330** | **-0.395 to -0.263** | **-0.226** | **-0.296 to -0.150** |
|  | → | NO_2_ | -0.050 | -0.115 to 0.016 | 0.029 | -0.035 to 0.094 |
|  | → | Noise | **-0.065** | **-0.130 to -0.002** | -0.038 | -0.103 to 0.028 |
| Income | → | MS | -0.028 | -0.092 to 0.036 | -0.057 | -0.123 to 0.009 |
|  | → | Dietary risk | -0.052 | -0.122 to 0.014 | -0.019 | -0.086 to 0.050 |
|  | → | Sedentary | 0.064 | -0.005 to 0.128 | **0.175** | **0.106 to 0.239** |
|  | → | Alcohol | 0.020 | -0.062 to 0.097 | **0.099** | **0.032 to 0.164** |
|  | → | Smoking | -0.024 | -0.104 to 0.057 | 0.073 | -0.031 to 0.148 |
|  | → | NO_2_ | -0.018 | -0.083 to 0.045 | -0.026 | -0.088 to 0.037 |
|  | → | Noise | -0.022 | -0.096 to 0.043 | -0.064 | -0.142 to 0.012 |
| NO_2_ | → | MS | 0.016 | -0.046 to 0.078 | 0.012 | -0.051 to 0.072 |
| Noise | → | MS | 0.053 | -0.013 to 0.122 | 0.048 | -0.013 to 0.110 |
| Dietary risk | → | MS | 0.059 | -0.005 to 0.124 | **0.131** | **0.077 to 0.185** |
| Sedentary | → | MS | **0.102** | **0.044 to 0.160** | **0.124** | **0.071 to 0.177** |
| Alcohol | → | MS | 0.047 | -0.013 to 0.111 | -0.016 | -0.069 to 0.035 |
| Smoking | → | MS | **0.127** | **0.056 to 0.193** | 0.015 | -0.048 to 0.076 |
| Results adjusted for age. MS: Metabolic syndrome score.  Model fit: Men: χ^2^(14) = 50.508, p<0.001, CFI = 0.938, RMSEA = 0.052, 90% CI RMSEA [0.037, 0.067]. Women: χ^2^(14) = 69.747, p<0.001, CFI = 0.924, RMSEA = 0.058, 90% CI RMSEA [0.045, 0.072]. | | | | | | |
